# Benefits of exercise intervention in reducing neuropathic pain

**DOI:** 10.3389/fncel.2014.00102

**Published:** 2014-04-04

**Authors:** John L. Dobson, Jim McMillan, Li Li

**Affiliations:** ^1^Department of Health and Kinesiology, Georgia Southern UniversityStatesboro, GA, USA; ^2^Key Laboratory of Exercise and Health Sciences, Ministry of Education, Shanghai University of SportShanghai, China

**Keywords:** peripheral neuropathy, neuropathic pain, exercise, inflammation, microglia, glycogen synthase kinase, cytokines

## Abstract

Peripheral neuropathy is a widespread and potentially incapacitating pathological condition that encompasses more than 100 different forms and manifestations of nerve damage. The diverse pathogenesis of peripheral neuropathy affects autonomic, motor and/or sensory neurons, and the symptoms that typify the condition are abnormal cutaneous sensation, muscle dysfunction and, most notably, chronic pain. Chronic neuropathic pain is difficult to treat and is often characterized by either exaggerated responses to painful stimuli (hyperalgesia) or pain resulting from stimuli that would not normally provoke pain (allodynia). The objective of this review is to provide an overview of some pathways associated with the development of peripheral neuropathy and then discuss the benefits of exercise interventions. The development of neuropathic pain is a highly complex and multifactorial process, but recent evidence indicates that the activation of spinal glial cells via the enzyme glycogen synthase kinase 3 and increases in the production of both pro-inflammatory cytokines and brain derived neurotropic factor are crucial steps. Since many of the most common causes of peripheral neuropathy cannot be fully treated, it is critical to understand that routine exercise may not only help prevent some of those causes, but that it has also proven to be an effective means of alleviating some of the condition’s most distressing symptoms. More research is required to elucidate the typical mechanisms of injury associated with peripheral neuropathy and the exercise-induced benefits to those mechanisms.

## Introduction

The etiology and pathology of peripheral neuropathy are complex and diverse. Additionally, the 108th U.S. Congress estimated that peripheral neuropathy affects at least 20 million U.S. Citizens, which is roughly 18% more Americans than are currently thought to be afflicted by coronary artery disease (Lloyd-Jones et al., 2009) and roughly 54% more than are currently thought to have some form of cancer (Howlader et al., 2012).

The defining characteristic of peripheral neuropathy is damage to the axons and/or myelin of one or more peripheral nerves, which often results in abnormal nerve conduction (e.g., low amplitudes or slow velocity) and/or spontaneous activity (Azhary et al., 2010). It is also typical for the deterioration to begin distally and then move progressively in a proximal or ascending manner. Yet, there are over 100 types of peripheral neuropathy (National Institute of Neurological Disorders and Stroke, 2012) and each differs not just in terms of etiology and the pattern of development, but also in terms of the nerves that are affected and the resulting symptoms. For example, damage occurring to autonomic nerves could lead to symptoms such as impaired: ventilation, blood pressure regulation, bladder control, digestion, sweating and tolerance to the heat However, it is more common for the condition to affect motor and sensory nerves, resulting in symptoms such as: muscle spasticity, muscle atrophy and strength loss; loss of sensations including vibration, touch, temperature and proprioception; and the presence of sensations such as tingling, burning and pain (Azhary et al., 2010; National Institute of Neurological Disorders and Stroke, 2012).

The complex symptomology of peripheral neuropathy is also attributed to the diverse etiology of the condition, which includes dozens of causes that are acquired, as well as some that are hereditary such as Refsum and Charcot-Marie-Tooth diseases (National Institute of Neurological Disorders and Stroke, 2012). The means by which neuropathy may be acquired include: physical injury (trauma); excessive exposure to toxins such as alcohol or heavy metals; deficiencies in vitamins including E, B6 and B12; or diseases such as Lyme and human immunodeficiency virus (Azhary et al., 2010). That said, it is estimated that as much as 30% of the cases of peripheral neuropathy are idiopathic (Smith and Singleton, 2006), whereas the majority of cases of the disease occur in conjunction with diabetes mellitus (National Institute of Neurological Disorders and Stroke, 2012).

**Figure 1:**
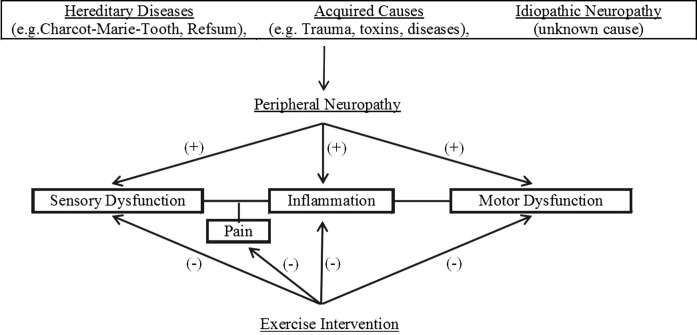
**Overview of potential benefits of exercise intervention on neuropathic pain**.

The primary purpose of this review is to discuss the potential benefits of exercise as an intervention for those with peripheral neuropathy. Figure 1 illustrates the flow of the discussions within this review. Because the mechanisms that underlie those benefits are not fully understood, we hope this review will help encourage further investigation to elucidate those mechanisms. To that end, we will also highlight a number of potentially important mechanisms by first summarizing key evidence concerning the development of both diabetic neuropathy and neuropathic pain. Although there are many other causes of peripheral neuropathy and the symptoms of the condition do not always include pain, the bulk of pertinent research has focused on these concerns and it is likely other manifestations of the condition share similar mechanisms of injury.

## Diabetic neuropathy

According to the Centers for Disease Control (Centers for Disease Control and Prevention, 2011), roughly 26 million American adults and children currently have diabetes mellitus, and 60–80% of those afflicted with the disease are expected to develop neuropathy. Among the collection of syndromes associated with peripheral neuropathy, the most common in diabetics is distal symmetrical sensorimotor polyneuropathy, which is often simply called diabetic neuropathy (Jack and Wright, 2012). A hallmark of diabetic neuropathy is symmetrical degeneration of small cutaneous sensory fibers, including myelinated A-δ and unmyelinated C-fibers, which are responsible for conveying cutaneous sensations like pain (Talbot and Couture, 2012). Accordingly, techniques that assess small fiber abnormalities, such as quantitative sensory testing (QST), intraepidermal nerve fiber (IENF) density and corneal confocal microscopy (CCM), can be effective means of detecting early diabetic neuropathy (Tavakoli et al., 2010). It is also not surprising that diabetes is the leading cause of painful neuropathy (Talbot and Couture, 2012) and that 30% of diabetics over the age of 40 have impaired sensation in their hands and/or feet (Centers for Disease Control and Prevention, 2011).

A crucial feature in the pathogenesis of diabetic neuropathy is hyperglycemia, and poor blood glucose control is recognized as a leading risk factor in the development of neurological problems (Jack and Wright, 2012; Talbot and Couture, 2012). One essential consequence of hyperglycemia is that it accelerates the production of advanced glycation end products (AGEs) in the tissues where damage occurs, including the peripheral nerves. AGEs are formed from at least two distinct pathways; one is the classic or glycation pathway and the other is the oxidative pathway. In the glycation pathway, a monosaccharide (e.g., glucose, fructose or galactose) and an amino group reversibly form an Amadori product (e.g., hemoglobin A1c), which may then be used to irreversibly generate an AGE (Huebschmann et al., 2006). The second pathway occurs in the presence of oxidative stress or reactive oxygen species (ROS), which become reactive dicarbonyl components that react with proteins, lipids and DNA to form AGEs (Jack and Wright, 2012). Hyperglycemia accelerates the production of AGEs from both the glycation and oxidative pathways (Huebschmann et al., 2006); consequently, it provides a key to understand why diabetics have extensive accumulations of AGEs in cells like myelinated and unmyelinated neurons, Schwann cells and microvessels (Jack and Wright, 2012).

Accumulated AGEs, along with other mechanisms that are enhanced with diabetes, such as oxidative stress, are likely significant contributors to the development of diabetic neuropathy (Andreassen et al., 2009; Nguyen et al., 2012). AGEs promote vascular disease (e.g., endothelial dysfunction, atherosclerosis and chronic vasoconstriction), which impairs neuron function by reducing blood flow (Huebschmann et al., 2006). AGEs also directly impair neuron function by modifying proteins in a manner that impairs vital cellular processes (e.g., regenerative capacity) and by initiating intracellular signaling cascades that increase the production of ROS and the expression of pro-inflammatory cytokines (Jack and Wright, 2012). For example, when AGEs interact with cell receptors for advanced glycation end products (RAGE), numerous intracellular signaling pathways are triggered that, in turn, may activate the DNA transcription factor NF-KB (e.g., nuclear factor kappa-light-chain-enhancer of activated B cells). NF-KB activation increases both the expression of pro-inflammatory cytokines interleukin 6 (IL-6) and tumor necrosis factor α (TNFα) and the expression of RAGE (Jack and Wright, 2012). The resulting enhanced AGE-RAGE interaction, NF-KB activation, and ROS production contribute to the development of persistent inflammation that damages peripheral neurons (Nguyen et al., 2012), causes pain (Bierhaus et al., 2004) and, more importantly, activates microglia (Talbot and Couture, 2012).

## Neuropathic pain and activated glia

Acute pain is informative and serves as a warning against impending or current tissue damage, whereas chronic pain is itself a pathological condition that serves no beneficial function. Chronic neuropathic pain is characterized by exaggerated responses to painful stimuli (hyperalgesia), pain resulting from stimuli that would not normally provoke pain (allodynia) and by abnormal pins and needles sensations. The transition from acute pain to chronic neuropathic pain is a highly complicated process that results in dysfunction throughout the pain transmission pathway, from the nociceptors (Ren and Dubner, 2010) to the dorsal root ganglion and spinal cord (Trang et al., 2011) to the thalamus (Fischer and Waxman, 2010) and, finally, the cerebral cortex (Zhuo et al., 2011). However, evidence strongly indicates that the activation of microglia in the dorsal horn of the spinal cord is a critical component in the initiation and maintenance of neuropathic pain (Scholz and Woolf, 2007; Leung and Cahill, 2010; Wen et al., 2011).

Microglial cells account for 5–12% of the total cells in the central nervous system and they typically exist in a relatively quiescent form, in which they contain long branching processes that monitor the physiological conditions of the surrounding environment (Perry et al., 2007). When microglia detect damage to surrounding neurons (e.g., peripheral nerve injury), they proliferate to form dense clusters, take on an amoeboid form and they begin to phagocytize cellular debris and foreign material. Under these conditions, the microglia are triggered by a number of complex signaling cascades, the details of which extend beyond the scope of this review (see Ren and Dubner, 2010; Scholz and Woolf, 2007 for reviews). However, the activation via adenosine triphosphate (ATP) interacting with P2 purinergic receptors has been identified as a particularly critical event. For example, a pivotal study by Tsuda et al. (2003) demonstrated the activation of P2X_4_ receptors in spinal microglia is necessary for tactile allodynia following nerve injury. Microglial activation via both ATP and tumor necrosis factor (TNF) leads to the phosphorylation (activation) of mitogen-activated protein kinases (MAPKs), including: extracellular signal-regulated kinases (ERK); Jun N-terminal kinases (JNKs); and, most notably, p38-MAPK. Chronic upregulation of P2X receptors and activation of MAPK signaling are considered essential components in the development and maintenance of neuropathic pain (Scholz and Woolf, 2007; Leung and Cahill, 2010; Trang et al., 2011; Wen et al., 2011) because they cause the following three significant actions. First, activated microglia synthesize and release brain-derived neurotrophic factor (BDNF), which encourages the growth and differentiation of neurons and synapses. Under pathological conditions, the BDNF released from microglia increases the excitability of nociceptive neurons (Ren and Dubner, 2010), reduces inhibitory γ-aminobutyric acid neurotransmission (Coull et al., 2005) and causes abnormal nociceptive processing that leads to hyperalgesia and allodynia (Kerr et al., 1999). Second, activated microglia release the pro-inflammatory cytokines TNFα., interleukin 1β (IL-1β) and IL-6. These inflammatory cytokines promote numerous pertinent actions associated with neuropathy and neuropathic pain, including: amplification of microglial activation (Zhuo et al., 2011); infiltration of phagocytic neutrophils (Nadeau et al., 2011); damage to Schwann cells (Xu and Yaksh, 2011); damage to axons and increases in nociceptor sensitivity (Schafers et al., 2003; Cunha et al., 2005); increased conductance in dorsal root ganglion neurons (Czeschik et al., 2008); and hyperactivity in dorsal horn neurons (Kawasaki et al., 2008; Ren and Torres, 2009). Third, activated microglia, along with the accumulation of pro-inflammatory cytokines and stimulation via purinergic receptors, cause spinal astrocytes to proliferate and became active (Maixner and Weng, 2013). Much like activated microglia, activated astrocytes promote (dorsal horn) neuron dysfunction by releasing BDNF (Ren and Dubner, 2010), IL-1β, IL-6 and TNFα (Beurel et al., 2010). However, because their activation is more prolonged than that of microglia, astrocytes are required to perpetuate the neuronal hyperexcitability, neurotoxicity and inflammation that characterizes neuropathic pain (Ji and Suter, 2007; Gosselin et al., 2010; Zhuo et al., 2011).

## Glial glycogen synthase kinase 3 regulates neuroinflammation

Glycogen synthase kinase 3 (GSK-3) mediates the addition of phosphate molecules onto more than 40 proteins in a variety of different pathways (typically inhibiting them), but it perhaps best known for its role in the regulation of glycogen synthesis. GSK-3 is a highly conserved protein kinase and its α- and β-isoforms are expressed in practically all mammalian tissues (Kaidanovich-Beilin and Woodgett, 2011). GSK-3 is a point of convergence of many different signaling pathways, it is known to regulate more than 20 transcription factors and it acts as a critical trigger both in the activation of glial cells and with the balance between pro-inflammatory and anti-inflammatory states (Beurel et al., 2010). In active microglia and astrocytes, GSK-3 promotes the release of IL-1β, IL-6 and TNFα and inhibits the release of anti-inflammatory cytokines like IL-10 (Beurel et al., 2010; Kaidanovich-Beilin and Woodgett, 2011; Maixner and Weng, 2013).

Although it is typically active in cells under resting conditions, GSK-3 is one of the few protein kinases that can be inhibited by extracellular factors via rapid phosphorylation of its serine residues. The extracellular signals that are known to inhibit GSK-3 include epidermal and platelet-derived growth factors (Stambolic et al., 1996; Shaw and Cohen, 1999), α_1A_, adrenergic receptor stimulation (Bailou et al., 2001) and insulin (Markuns et al., 1999). Given the typical fluctuations in insulin levels, and because the hormone is particularly relevant to this review (i.e., diabetic neuropathy is the leading cause of neuropathy), it is worth noting both that insulin uses the Akt/protein kinase B pathway to phosphorylate GSK-3 (Cross et al., 1995) and that insulin’s effect on GSK-3 is altered by angiotensin II (Diamond-Stanic and Henriksen, 2010) and α_1A_ (Bailou et al., 2001) stimulation. That said, it is clear that significant/prolonged inactivation of GSK-3 in microglia and astrocytes shifts balance of secreted cytokines from pro-inflammatory to anti-inflammatory (Beurel et al., 2010). For example, pharmacological inhibition of GSK-3 activity in activated glial cells has been shown to reduce elevated secretions of TNFα and IL-1β (Wang et al., 2010; Green and Nolan, 2012), to elevate decreased secretions of IL-10 (Green and Nolan, 2012) and to reduce neuropathic pain (Mazzardo-Martins et al., 2012).

## Exercise and peripheral neuropathy

Most cases of peripheral neuropathy cannot be fully treated because the underlying cause is either unknown (i.e., idiopathic neuropathy) or it cannot be cured (e.g., inherited and diabetic neuropathies). Therefore, the major goal associated with the treatment of most forms of peripheral neuropathy is to control or ameliorate the troublesome symptoms (National Institute of Neurological Disorders and Stroke, 2012). To summarize the above sections, the symptoms that typify peripheral neuropathy are numbness, reduced proprioception, weakness, poor balance and, in particular, allodynia and hyperalgesia,; the mechanisms that underlie those symptoms may include: hyperglycemia and AGEs (diabetic neuropathy), GSK-3 and glial activation, and elevated levels of the pro-inflammatory cytokines TNFα, IL-1β and IL-6.

As we (Li and Hondzinski, 2012) have observed before, routine exercise can be a beneficial addition to medical and pharmaceutical treatments for people with peripheral neuropathy. According to the U.S. Surgeon General (U.S. Department of Health and Human Services, 1996) and the American College of Sports Medicine (American College Sport Medicine, 2010), the myriad relevant preventative benefits of routine exercise include: enhanced macro- and micro-vascular health (e.g., better endothelial function, reduced vasoconstriction and enhanced blood flow); reduced risk of hypertension, atherosclerosis and numerous cardiovascular diseases; decreased production of ROS and increased anti-oxidant defenses; reduced risks of certain types of cancer; increased muscle strength and cardiorespiratory endurance. With specific regard to the most common cause of peripheral neuropathy (i.e., diabetes), exercise is also well-known to reduce: blood glucose levels, the formation of Amadori products (Balducci et al., 2010; Ahn and Song, 2012; Kluding et al., 2012), the accumulation of AGEs (Boor et al., 2009; Yoshikawa et al., 2009; Kotani et al., 2011) and the risk of developing type II diabetes and metabolic syndrome (American College Sport Medicine, 2010; Li and Hondzinski, 2012).

In contrast to the many benefits listed above, the relationships between exercise and both glia activation and GSK-3 activity have not been extensively studied and remain less clear. For example, although a number of studies (Markuns et al., 1999; Sakamoto et al., 2003; Aschenbach et al., 2006) have demonstrated that a single bout of exercise can temporarily phosphorylate (inactivate) GSK-3 in rodents, the evidence in humans has been more inconsistent (Nielsen and Richter, 2003; Sakamoto et al., 2004). Furthermore, a recent study by Manabe and co-workers (Manabe et al., 2013) found that chronic exercise training actually resulted in prolonged increases in GSK-3β activity in rodents. However, it is very important to point out that, in all of the studies just mentioned, GSK-3 activity was measured in enzymes that were within the skeletal muscles that had been involved in the exercise. To the best of the authors’ knowledge, only one study (Bayod et al., 2011) has investigated the effect of exercise on GSK-3 activity in cells located within the central nervous system (i.e., where glia are located), and that study found that training resulted in prolonged reductions in GSK-3 activity in rodent hippocampal cells. It is also important to note that the effects of exercise on skeletal muscle GSK-3 activity are thought to be triggered specifically by tension within the muscle (Sakamoto et al., 2003). Therefore, whatever effects exercise has on spinal glia would have to be initiated in a different manner and, consequently, would likely involve different signaling pathways. While there is some evidence that exercise training alters the activity of astrocytes (Bernardi et al., 2013) and microglia (Cobianchi et al., 2010), the relationship between exercise, glial activation and neuropathic pain requires much more investigation. That said, future studies in this area should take into consideration the following two sets of relevant evidence. First, norepinephrine, which is increased systemically during exercise via enhanced sympathoadrenal activity, may inactivate GSK-3β (Bailou et al., 2001) and has been shown to reduce both the proliferation of microglia and the release of pro-inflammatory cytokines from microglia and astrocytes (O’Donnell et al., 2012). Second, contracting muscles secrete cytokines (i.e., myokines) that tend to have more anti-inflammatory properties (Moldoveanu et al., 2001; Brandt and Pedersen, 2010; National Institute of Neurological Disorders and Stroke, 2012), and routine exercise helps prevent and/or reduce low-grade systemic inflammation (Smith et al., 1999; Mathur and Pedersen, 2009; Phillips et al., 2010; Gano et al., 2011; Hopps et al., 2011; Irwin and Olmstead, 2012).

Aside the preventative and more general benefits of exercise just summarized, it is clear that routine exercise may be a highly effective means of enhancing the recovery from, and/or reducing some of the distressing symptoms associated with, peripheral neuropathy. Among the dozens of pertinent studies, a substantial number focused on the effect of either strength-stability exercises (e.g., Tai Chi) or aerobic exercise (e.g., walking) in humans with diabetic peripheral neuropathy. All of the remaining studies examined the effect of aerobic exercise (e.g., treadmill running) in rodents either with diabetic peripheral neuropathy or following nerve trauma that was elicited via acid, crushing or cutting. Because the mechanisms investigated and benefits identified with both of those subgroups of neuropathic rodents were the same, the results of both sets of rodent studies will be generalized in the following discussion.

Beginning with the components that are most directly affected by peripheral neuropathy, routine exercise has been shown to both preserve and promote the function of the peripheral nerves. For example, Balducci et al. (2006) compared people with diabetes following 4 years of aerobic exercise training vs. control and found that significantly fewer of those in the exercise group developed motor (0% vs. 17%, respectively) and sensory (6% vs. 30%) nerve dysfunction. Among those who have already suffered nerve damage, the evidence from rodent studies indicates that aerobic exercise promotes peripheral nerve regeneration following injury (Ilha et al., 2008; Malysz et al., 2010; O’Donnell et al., 2012). Additionally, routine exercise has been shown to both enhance peripheral nerve conduction velocity (Balducci et al., 2006; Hung et al., 2009) and increase IENF branching (Kluding et al., 2012) and density (Smith et al., 2006) in diabetic humans. On a related note, it is possible that exercise training could elicit favorable adaptions in the nervous system via plasticity mechanisms and by retraining neural pathways, but, to the best of the authors’ our knowledge, this has not been investigated.

The most frequently cited relevant benefit of exercise has been its positive effects on sensation, and most notably neuropathic pain. In rodents, aerobic exercise training has been shown to delay the onset of diabetic pain (Chen et al., 2013) and tactile hypersensitivity (Shankarappa et al., 2011), and to also reduce mechanical allodynia (Sharma et al., 2010; Bobinski et al., 2011; Stagg et al., 2011; O’Donnell et al., 2012; Cobianchi et al., 2013) and hyperalgesia (Hutchinson et al., 2004; Chen et al., 2013; Sluka et al., 2013) following injury. Similarly, routine exercise not only alleviates neuropathic pain in humans, but it has also been shown to increase cutaneous (i.e., plantar) sensation (Li and Manor, 2010) and the ability to perceive vibrations (Balducci et al., 2006). Those sensory benefits, along with improvements in trunk (Song et al., 2011) and ankle proprioception (Xu et al., 2004), likely contribute to the positive effect that exercise training, and in particular Tai Chi, has on balance (Orr et al., 2006; Morrison et al., 2010; Song et al., 2011; Ahn and Song, 2012; Akbari et al., 2012; Li and Hondzinski, 2012) and functional mobility (Orr et al., 2006; Li and Manor, 2010; Song et al., 2011) in those with peripheral neuropathy.

As to the physiological explanations for the benefits just described, few studies have investigated the effects of exercise on the typical mechanisms of injury with peripheral neuropathy that were discussed above. For example, routine exercise has been shown to decrease levels of pro-inflammatory cytokines in diabetic humans (Dekker et al., 2007; Balducci et al., 2010) and rats (Teixeira de Lemos et al., 2009), but exactly how exercise-induced changes in cytokines affect those with peripheral neuropathy has yet to be fully explored. As to BDNF, studies have demonstrated that exercise training tends to elevate the expression of BDNF in both the CNS (Gomez-Pinilla et al., 2012; Rothman et al., 2012) and skeletal muscles (Gomez-Pinilla et al., 2012) and that those increases may be beneficial to cognition and the health of the brain (Rothman et al., 2012). However, as discussed above, excessive levels of BDNF in sensory locations like the dorsal horn and dorsal root ganglion are associated with abnormal nociceptive processing and the development of neuropathic pain. That said, BDNF does play an important role in the regeneration of injured peripheral axons (Wilhelm et al., 2012), and there is some evidence exercise increases BDNF expression in motor, but not sensory, neurons following peripheral nerve injury (Keeler et al., 2012). Furthermore, Cobianchi et al. (2013) demonstrated that aerobic exercise training reduced both levels of BDNF in the dorsal root ganglion and neuropathic pain in rats following peripheral nerve injury. Similarly, Detloff et al. (2014) very recently showed that aerobic exercise can normalize spinal levels of glial cell-derived neurotrophic factor (GDNF), prevent excessive sprouting of pain afferents and reduce tactile allodynia in rats following spinal cord injury.

To summarize, the relationships between exercise and cytokines, BDNF, glia and GSK-3 in those with peripheral neuropathy have yet to be elucidated and must continue to be studied. Additionally, a number of additional important mechanisms have recently been identified and, although it is not clear how they interact with those discussed above, these novel mechanisms have been linked to pertinent exercise-induced benefits and should therefore continue to be explored.

The first such novel mechanism is neurotrophin-3 (NT-3), which is known to promote the survival and differentiation of existing neurons and to encourage the growth of new synapses and neurons. Aerobic exercise training has been shown to increase the expression NT-3 in rodents in the spinal cord (Gómez-Pinilla et al., 2001) and skeletal muscles of both diabetics (Li et al., 2012) and those recovering from peripheral nerve injury (Hutchinson et al., 2004). Those observed exercise-induced elevations of NT-3 were associated with increases in peripheral nerve conduction velocity (Li et al., 2012) and reductions in neuropathic pain (Sharma et al., 2010).

A second mechanism that has recently been shown to provide pertinent benefits is endogenous opiates such as met-enkephalin and β-endorphin. The production of these opiates is enhanced during exercise, and their action promotes analgesia and feelings of well-being (e.g., “runners high”). It is therefore not surprising that exercise–induced increases in endogenous opiates accompany reductions in neuropathic pain in rats with diabetes (Shankarappa et al., 2011) and following nerve injury (Bement and Sluka, 2005; Sluka et al., 2013). Furthermore, Stagg et al. (2011) demonstrated not only that aerobic exercise training reduced neuropathic pain, but that the analgesic effect depended on the stimulation of central opioid receptors.

The third and final mechanism that is affected by exercise and helps to counteract the dysfunction associated with peripheral neuropathy is heat shock protein 72 (HSP72). HSP72 is a member of a family of heat shock proteins (i.e., the 70 kilodalton family) that is known to fold proteins, including refolding denatured proteins, and is needed to help protect cells from stress (Yamada et al., 2008). The expression of HSP72 tends to decrease with diseases like diabetes (Atalay et al., 2004) but aerobic exercise enhances its expression throughout the body, including in the peripheral nerves and spinal cord (Chen et al., 2013) and in skeletal muscles (Ogata et al., 2009). HSP72 has been shown to increase tolerance to inflammatory cytokines, and there is some evidence that it may reduce their secretion by inactivating the NF-KB pathway (Yamada et al., 2008). Additionally, a very recent study by Chen et al. (2013) demonstrated that exercise-induced increases in HSP72 delayed the onset hyperalgesia and mechanical allodynia in diabetic rats.

There are many studies that focus exercise induced changes due to neuroplasticity. That is beyond the scope of this review. Please note this limitation of current review and seeking in depth information in regarding exercise and neuroplasticity among people with neuropathic pain elsewhere.

## Conclusions

There are a great many different causes of, and symptoms associated with, peripheral neuropathy. However, many of the most common manifestations of the condition involve chronic neuroinflammation and neuropathic pain, and current evidence indicates the mechanisms underlying those manifestations may include: hyperglycemia and AGEs (diabetic neuropathy), GSK-3 and glial activation and elevated levels of both BDNF and the pro-inflammatory cytokines TNFα, IL-1β and IL-6. Although there is no cure for most forms of peripheral neuropathy, routine exercise may either prevent or delay the onset of the some of the most common causes. As to the studies that investigated the treatment benefits of exercise training, nearly all focused on diabetic neuropathy or on animals following spinal cord/nerve injury induction, and so their findings cannot generalize to all forms of peripheral neuropathy. Nevertheless, the models used in those investigations included the most common cause of peripheral neuropathy (i.e., diabetes); they studied the characteristics that are central to all forms of the condition (e.g., nerve damage and dysfunction); and the benefits they reported are relevant to the symptoms of nearly all manifestations of the condition. Those benefits of exercise training include improvements in nerve function, reductions in neuropathic pain, reductions in other types of sensory dysfunction (e.g., numbness) and improvements in both static and dynamic functional mobility in those with peripheral neuropathy. Therefore it may be conclude that exercise training can be an effective intervention for many of those symptoms associated with peripheral neuropathy.

Future research in this area should continue to investigate the relationship between both glial GSK-3 activity and levels of TNFα, IL-1β and IL-6 and exercise-induced reductions in neuron dysfunction, neuropathic pain and neuroinflammation. Future studies should also continue to investigate if the specific nature of the exercise- induced benefits varies with the mode of training (aerobic vs. strength). The authors hypothesize that a combined aerobic and strengthening activity such as Tai Chi will promote a relatively broad range of benefits to those with peripheral neuropathy.

## Author contributions

Li Li and Jim McMillan initialized the concept of the paper. John L. Dobson wrote the first draft and made the revisions based on comments from Li Li and Jim McMillan. All authors participated final revision of the manuscript.

## Conflict of interest statement

The authors declare that the review was written in the absence of any commercial or financial relationships that could be construed as a potential conflict of interest.
